# Larval habitats and species diversity of mosquitoes (Diptera: Culicidae) in West Azerbaijan Province, Northwestern Iran

**DOI:** 10.1186/s12898-020-00328-0

**Published:** 2020-11-19

**Authors:** Mojtaba Amini, Ahmad Ali Hanafi-Bojd, Ali Ahmad Aghapour, Ali Reza Chavshin

**Affiliations:** 1 Department of Medical Entomology and Vector Control, School of Public Health and Social Determinants of Health Research Center, Urmia University of Medical Sciences, Urmia, Iran; 2 Student Research Committee, Urmia University of Medical Sciences, Urmia, Iran; 3 Department of Medical Entomology and Vector Control, School of Public Health, Tehran University of Medical Sciences, Tehran, Iran; 4 Department of Environmental Health Engineering, School of Public Health, Urmia University of Medical Sciences, Urmia, Iran; 5 Social Determinants of Health Research Center, Urmia University of Medical Sciences, Urmia, Iran

**Keywords:** Mosquito larval habitats, Oviposition sites, Species diversity

## Abstract

**Background:**

The characteristics of a larval habitat is an important factor which affects the breeding pattern and population growth of mosquitoes Information about the larval habitat characteristics and pupal productivity can be utilized for the surveillance of the level of population growth, species diversity, and preferred breeding sites of mosquitoes, which are important aspects of integrated vector control. In the present study, mosquito larvae were collected from 22 natural habitats in five counties of the West Azerbaijan Province in the Northwest of Iran during May–November 2018. Physicochemical characteristics of the habitats were investigated. These included alkalinity, chloride (Cl) content, water temperature (°C), turbidity (NTU), Total Dissolved Solids (TDS) (ppm), Electrical Conductivity (EC) (μS/cm), and acidity (pH). The index of affinity between the collected species was calculated using Fager & McGowan test.

**Results:**

A total of 2715 specimens were collected and identified. Seven different species belonging to four genera were identified in our study sites. The species included, *Culex pipiens* Linnaeus 1758, *Culex theileri* Theobald 1903*, Culex mimeticus* Noé 1899, *Culex modestus* Ficalbi 1947*, Culiseta longiareolata* Macquart 1838*, Anopheles maculipennis* Meigen 1818complex, and *Aedes caspius* Pallas 1771. There was a significant difference in chloride content and water temperature preferences among the different species (P < 0.05). Also, there was no significant difference in pH, Alkalinity, Turbidity, TDS, and EC preferences among the different species (P > 0.05). The affinity between the pair of species *Cx. mimeticus*/*Cs. longiareolata* was 0.526. There was no affinity between other pairs of species or the affinity was very weak.

**Conclusions:**

The physicochemical and biological characteristics of mosquito larval habitats play an important role in zoning of areas suitable for breeding and distribution. Surveillance of these characteristics can provide valuable information for entomological monitoring of mosquito vectors and for designing targeted control programs. Also, further studies should be undertaken in a wider geographical area, taking into account the complex characteristics of the physicochemical and ecological factors of the study area and their interaction with various mosquito species.

## Background

The breeding pattern and population growth of medically important mosquitoes are affected by the physicochemical characteristics of their habitats. Breeding pattern and population growth surveillance is essential in designing mosquito control programs [1]. Monitoring mosquito larval habitats and population growth pattern, in the form of studies to identify the characteristics of the habitats, can provide valuable information on mosquitoes’ population growth levels, species diversity, and breeding sites for Integrated Vector Management (IVM) [2].

Vector control programs rely on the thorough knowledge of the ecology and population dynamics of mosquito species, as well as the epidemiology of mosquito-borne diseases. Therefore, research on vector habitats must be stepped up so that mosquito control programs could be carried out on sound bases [3].

The factors which affect mosquitoes’ oviposition site selection, also play a crucial role in larval density and species composition [4, 5]. In recent years, the suitable temperature [6, 7] and pH range [6] for the presence and abundance of larval species of mosquitoes have been studied and identified. Also, a direct correlation between the distribution of medically important mosquito species and physicochemical properties of their habitats, including temperature, ammonia, nitrate, pH, dissolved oxygen, and salinity has been reported by previous studies [8, 9].

In different regions of the world, the influence of physicochemical properties of mosquito larval habitats on the production of emergent mosquitoes [10], their body size [10], and embryonic development and adult fitness [11] have also been studied. Moreover, it has been reported that the spatiotemporal patterns of mosquito production could be influenced by variation in the properties of breeding sites, which are important determinants of growth and survival of larval populations. Thus, the identification of these factors is essential for developing control strategies for mosquitoes [10].

Worldwide, as well as in Iran, there has been greater emphasis on the study of malaria vectors, however, most of the studies have focused on the influence of these physicochemical factors on malaria vectors [4, 12–14].

Only few studies have focused on the influence of these factors on mosquito larvae. In a previous study which investigated the effects of physicochemical characteristics of larval habitat waters on a broader range of mosquito genera, there was no significant difference in temperature, pH, turbidity, electrical conductivity (*EC*), total dissolved solids (*TDS*), alkalinity, total hardness, calcium, chloride, fluoride, nitrite, nitrate, phosphate, and sulphate content of the larval habitat water among the different mosquitoes species in Qom Province in the central part of Iran [15]. In another study, a positive correlation between the larval abundance of *Cx. pipiens* and the physicochemical characteristics of the larval habitat including EC, alkalinity, total hardness, and chloride content. However, the negative correlation between these characteristics and larval abundance was not significant [16].

Also, the environmental changes driven by urbanization and agricultural and industrial projects affect mosquito species’ diversity. Biodiversity and related important changes in geographic ranges of mosquitoes are of medical and veterinary importance, because any distributional shifts could alter and expand the range of mosquito-borne diseases [17–19].

The West Azerbaijan Province in the northwestern Iran is an important biogeographic region which shares a common border with four countries; Armenia, Azerbaijan, Iraq, and Turkey. These countries are endemic areas for a variety of mosquito species due to the presence of various environmental and geographical conditions favorable for the breeding of mosquitoes [20–23]. Consequently, there is a high diversity of mosquito species and spread of mosquito-borne diseases in the West Azerbaijan Province [24–26]. Also, this province is well-known for its abundant water resources and wetlands for migratory birds from around the world [27, 28].

By identifying the characteristics of larval habitats and species diversity of mosquitoes, the prospect and possibility of mosquito borne diseases in a particular geographical area could be determined with higher precision, and consequently, integrated vector management can be implemented with guaranteed success. The specific geographical location of the West Azerbaijan Province, its remarkable climatic diversity and abundant water resources [27], as well as the presence of a variety of mosquitoes and history of mosquito-borne diseases in this area has attracted the attention of public health research in recent years. The present study aimed to investigate (1) some of the physicochemical properties of mosquito larval habitats (temperature, pH, turbidity, Chloride, Alkalinity, TDS and EC), (2) possible correlation between the physicochemical parameters and the presence of mosquito species, and (3) species diversity, co-occurrence and affinity index between pairs of mosquitoes species. The results of this study would be useful in determining the environmental and biological factors of the larval habitats of mosquito vectors.

## Methods

### Study area

**Fig. 1:**
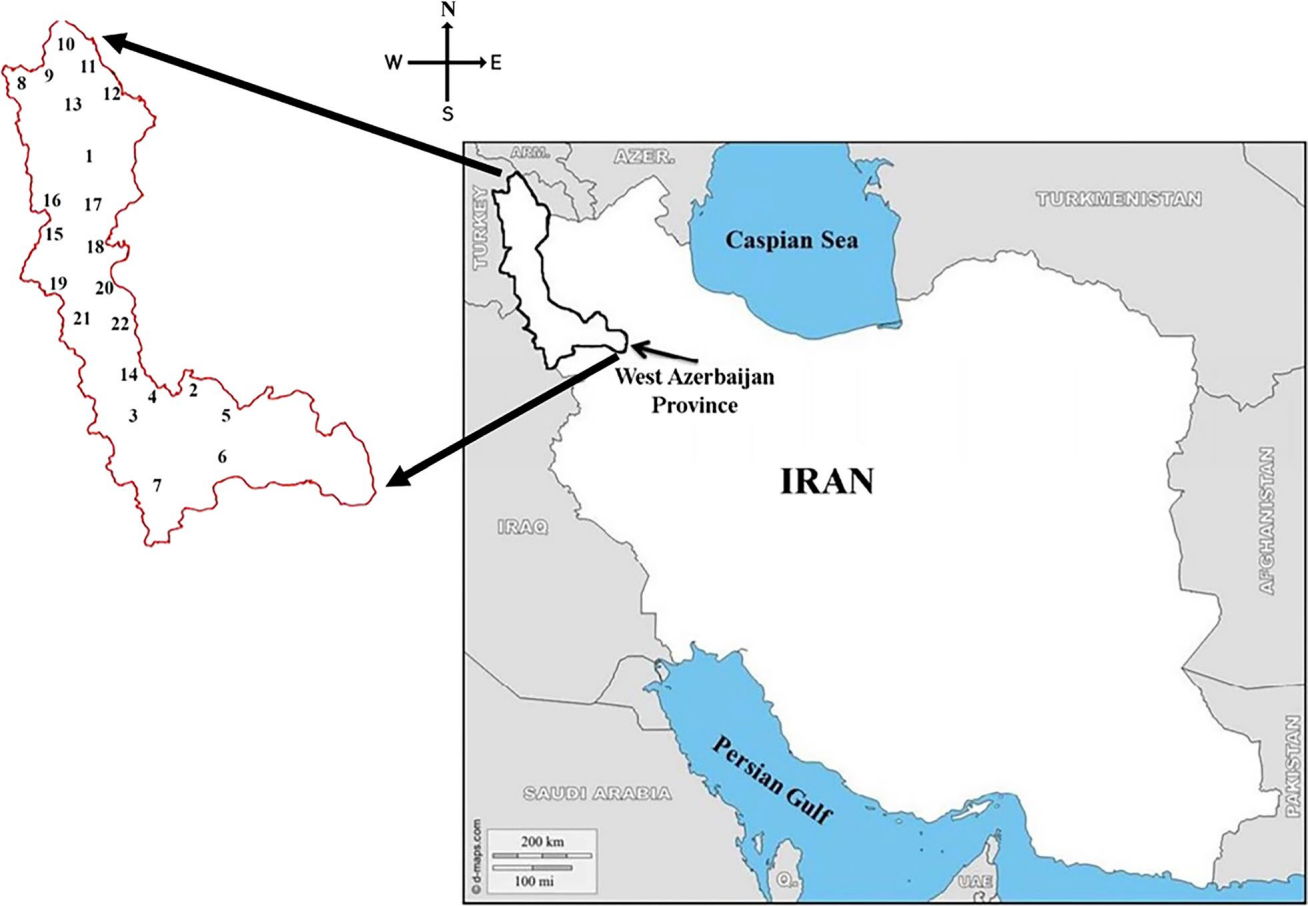
Geographical location of West Azerbaijan Province in northwestern Iran and common border with different countries. Sampling sites: 1. Hashiyeh Rood, 2: Hajib Khosh, 3: Kanibrazan Wetland, 4: Khor Khoreh, 5: Gapis, 6: Beytas, 7: Mahabad, 8: Milan, 9: Sangar, 10: Sangar2, 11: Keshmesh Tappeh, 12: Deimgeshlag, 13: Glik Gadim, 14: Yadegarloo, 15: Silvana, 16: Gojar, 17: Kooraneh, 18: Ghahramanloo, 19: Mavana, 20: Shaharchay Dam, 21: Talebin, 22: NAzloo(Original basic map has been prepared from https://www.d-maps.com/)

The West Azerbaijan Province is located in the northwest of Iran between latitudes 39° 46´- 35° 71 58´ N and longitudes 44° 3´- 47° 23´ E. This province shares border with 4 neighboring countries: Armenia, Azerbaijan, Iraq, and Turkey (Fig. 1). This area is characterized by forest steppe with various climates, including Mediterranean hot summer climate, coastal Mediterranean climate, and the Cold Winter climate. It also has various geographical regions such as mountainous areas near the Iraq–Turkey border, plains near the Aras and other rivers, and the Urmia Lake coastline.

### Mosquito collection and identification

Larvae were collected using the probability based sampling method [29] from natural fixed habitats in different regions of the province (with variation in climate, habitat type, etc.), so that the results can be generalized to a wide range of habitats in the province. The selected habitats included ground pools, wetlands, stream edges, riverbeds, and river edges. The standard dipping method [30] was used for collecting specimens in 22 localities of five counties on a monthly basis between May and November 2018 (Fig. 1 andTable 1). Larvae were preserved in lactophenol. Microscope slides were prepared using de Faure’s medium. Third and 4th instar larvae were identified using the Iranian mosquitoes’ identification keys based on morphological characteristics [31].

**Table 1:** The geographical properties of sampling localities

County	Location	Longitude	Latitude	Altitude	Type of larval habitat
Khoy	Hashiyeh Rood	45.063147	38.571167	1058	river-side
Mahabad	Hajib Khosh	45.797183	36.907883	1288	canal
	Kanibrazan Wetland	45.764014	36.963683	1279	wetland
	Khor Khoreh	45.7235	36.987533	1282	River-side
	Gapis	45.747953	36.93805	1286	Ground pool
	Beytas	45.694267	36.67645	1396	stream edge
	Mahabad	45.722667	36.776683	1310	dam
Makoo	Milan	44.427261	39.340794	1373	canal
	Sangar	44.432683	39.317183	1349	swamp
	Sangar2	44.435567	39.3112	1342	Stream edge
	Keshmesh Tappeh	44.400933	39.333514	1385	Water reservoir
	Deimgeshlag	44.798697	39.624883	797	Rock-pool
	Glik Gadim	44.667667	39.712636	807	swamp
Nagadeh	Yadegarloo	45.528386	37.038217	1284	wetland
Urmia	Silvana	44.851419	37.428667	1577	River-side
	Gojar	44.833728	39.487847	1736	Rock-pool
	Kooraneh	44.687583	37.7234	1563	swamp
	Gahramanloo	45.152514	37.6221	1395	wetland
	Mavana	44.796433	37.566583	1617	Stream edge
	Shaharchay Dam	44.986283	37.4952	1433	dam
	Talebin	44.833983	37.54025	1608	Water reservoir
	Nazloo	44.985333	37.652683	1360	canal

### Physicochemical analyses

Water samples were collected from the 22 larval habitats for at least 6 times during the study period (once a month), and the water samples were kept in 1.5 L appropriately labeled bottles. The bottles were placed inside an icebox and were transferred to laboratory. Alkalinity, chloride (Cl), water temperature, turbidity, Total Dissolved Solids (TDS), Electrical Conductivity (EC), and acidity (pH) were selected because of previous scientific reports about their correlation with the presence and abundance of different species of mosquitoes and the possibility of measuring them.

The chemical factors, including alkalinity, chloride (Cl), were analyzed based on mg/l using standard methods [32]. The SI units for the other physicochemical parameters that were measured are as follow: water temperature (°C), turbidity (NTU), Total Dissolved Solids (TDS) (ppm), Electrical Conductivity (EC) (μS/cm), and acidity (pH). Water temperature and pH were measured on-site using a thermometer and pH probe (HANNA), respectively, and the other parameters were measured in the laboratory. Turbidity was measured using a turbidimeter device (2100P Portable Turbidimeter at Hach) and EC was measured using a conductometer device (Sension EC5 at Hach). Alkalinity and chloride content were determined using direct titration techniques. All analyses were performed according to standard methods [32].

### Statistical analyses

The mean and standard deviation of each physicochemical parameter were calculated for each breeding site. The assumption of normality distribution was tested using the Kolmogorov–Smirnov test. Based on the results, the one-way ANOVA and Kruskal–wallis tests for variables with normal and non-normal distribution were used respectively for determination of probable statistical significant difference of means at 0.05 significance level, among the breeding sites. Then Post-hoc analysis were used to determine the significant difference between different points (in pairs). Dunn-Bonferroni and Ferroni tests were used for factors with non-normal and normal distribution, respectively.

The Canonical Correspondence Analysis (CCA) is used to elucidate the effects of environmental variables on the presence/abundance of species [33]. In the present study, it was used to determine the response of species composition to the studied environmental variables (CCA) [34].

### Indices of affinity

The index of affinity was calculated using Fager & McGowan test [35] to find the affinity between pairs of Culicidae species occurring in the same habitats, based on the following formula:

$$\left[ {{\text{J}}/\left( {\text{NANB}} \right)\raise.5ex\hbox{$\scriptstyle 1$}\kern-.1em/ \kern-.15em\lower.25ex\hbox{$\scriptstyle 2$} } \right] \, {-}{ 1}/ 2\left( {\text{NB}} \right) \, \raise.5ex\hbox{$\scriptstyle 1$}\kern-.1em/ \kern-.15em\lower.25ex\hbox{$\scriptstyle 2$}$$

$$I\;\text{ = }\;\left[ {\frac{J}{{\left( {nA\text{ + }\;nB} \right)^{{1\text{/}2}} }}} \right]\;\text{ - }\left[ {\frac{1}{{2\left( {nB} \right)^{{1\text{/}2}} }}} \right]$$

where the number of joint occurrences (J); the total number of occurrences of species A (nA); the total number of occurrences of species B (nB), and species are assigned to the letters so that nA < nB. The expressions equal to or higher than 0.5 were considered strong affinity.

Fager and McGowan [35] chose this cutoff because they felt that for species to be grouped together as co-occurring species, they should be found together in more than “half” their recorded occurrences. On the other hand, it is assumed that species found together in more than 50% of collection sites most probably have the same environmental needs. In other words, when one of the co-occurred species is found, it is likely that the second species is also present in the same habitat.

To evaluate the significance of this index, the “t” test was applied, considering an arbitrary significance level of 5%. The “t” test was calculated according to the formula:

$$t\;\text{ = }\;\left[ {\left[ {\left( {nA\;\text{ + }\;nB} \right)\left( {2J\text{ - }\;1} \right)\text{/}\left( {2nA\;nB} \right)} \right]\text{ - }\;1} \right]\;\left[ {\left( {nA\;\text{ + }\;nB} \right)\text{ - }\;1} \right]^{1/2}$$

$$t\;\text{ = }\;\left[ {\frac{{\left( {nB\text{ + }\;nB} \right)\;\left( {2J\text{ - }\;1} \right)}}{{\left( {2nAnB} \right)}}\text{ - }\;1} \right]\;\left[ {\sqrt {\left( {nA\;\text{ + }nB\;\text{ - }\;1} \right)} } \right]$$

[36, 37].

## Results

A total of 2715 mosquito specimens were collected. Seven different species belonging to four genera were identified in our study sites, including *Culex pipiens* Linnaeus 1758 (n: 877, 32.3%), *Culex theileri* Theobald 1903, (n: 515, 18.9%) *Culex mimeticus* Noé 1899, (n: 569, 20.9%), *Culex modestus* Ficalbi 1947, (n: 2, 0.07%), *Culiseta longiareolata* Macquart 1838, (n: 29, 2.17%) *Anopheles maculipennis* Meigen 1818 complex (n: 263, 9.7%), and *Aedes caspius* Pallas 1771 (n: 430, 15.8%).

*Cx. pipiens* was collected from 15 (68.2%) out of the 22 collection sites. The second most distributed species was *Cx. theileri*. This species was found in 13 (59.1%) collection sites. The distribution of the other species was as follows: *An. maculipennis complex,* 10 (45.5%) sites; *Ae. caspius,* 6 (27.3%) sites; 5 (22.7%) collection sites for both *Cx. mimeticus* and *Cs. longiareolata*; and *Cx. modestus,* 1 (4.5%) site. In total, anopheline larvae were found in 10 sites (45.5%), whereas culicinae larvae inhabited 22 (100%) sites (Table 2).

**Table 2:** Result of chemical analysis of mosquitoes’ breeding places and the number collected of mosquitos’ specimens in west Azerbaijan province in 2018

County		Locality																							
	Khoy	Mahabad						Makoo						Nagadeh	Urmia										
Location	Hashiyeh Rood	Beytas	Gapis	Hajib Khosh	Kanibrazan Wetland	Khor Khoreh	Mahabad	Deimgeshlag	Glik Gadim	Keshmesh Tappeh	Milan	Sangar 1	Sangar 2	Yadegarloo	Gahramanloo	Gojar	Kooraneh	Mavana	Nazloo	Shaharchay Dam	Silvana	Talebin	Mean(n = 22)	SD	*P Value* (of Kruskal–wallis test)
pH	8	7	7	7	7	7	7	7	7	8	8	8	9	7	7	7	8	7	7	7	7	7	7.32	0.57	0.000
Temperature	27	25	25	25	25	25	25	28	27	27	27	26	27	23	23	23	23	23	23	23	23	23	24.82	1.76	0.000
Turbidity	10	9	2	4	1	2	3	7	1	6	5	1	1	5	1	2	6	1	1	0	0	1	3.14	2.93	0.0001
Chloride	609	187	14	173	226	295	105	102	179	51	19	19	48	57	224	20	59	5	45	1	21	23	112.82	40.32	0.0001
Alkalinity	240	210	80	267	180	217	290	490	125	324	160	290	516	310	490	304	604	316	170	136	324	34	276.23	146.03	0.000
TDS	145	749	218	145	993	149	769	142	592	808	925	660	125	440	944	414	408	395	445	174	421	435	477.09	288.38	0.000
EC	242	1248	363	242	1655	249	1281	236	987	1346	1541	1100	208	733	1574	690	680	659	741	290	701	725	795.05	480.55	0.000
*Cx. pipiens*	6	28	4	136	13	50	33	20	26	150	0	40	0	0	24	15	21	0	311	0	0	0			
*Cx. theileri*	18	39	7	0	0	0	18	0	0	94	15	64	0	23	0	19	0	32	27	10	0	149			
*Cx. mimeticus*	0	0	0	0	0	0	0	0	0	0	0	0	0	70	0	25	0	140	0	20	240	74			
*Cx. modestus*	0	0	0	0	0	2	0	0	0	0	0	0	0	0	0	0	0	0	0	0	0	0			
*Cs. longieraolata*	0	0	0	0	0	0	0	0	0	0	0	0	0	19	0	7	6	24	0	3	0	0			
*An. maculipennis*	0	0	0	0	0	0	2	0	0	0	30	26	36	10	0	7	8	13	0	11	120	0			
*Ae. caspius*	3	3	0	0	0	5	0	190	31	0	0	98	100	0	0	0	0	0	0	0	0	0			
Sum N (%)	27 (0.9)	70 (2.57)	11 (0.40)	136 (5)	13 (0.47)	57 (2.09)	53 (1.95)	210 (7.7)	57 (2.09)	244 (8.9)	45 (1.65)	228 (8.39)	136 (5)	122 (4.49)	24 (0.88)	73 (2.68)	35 (1.28)	209 (7.69)	338 (12.44)	44 (1.62)	360 (13.25)	223 (8.21)	**2715**		

The physicochemical parameters of the different collection sites and the results of Kruskal–wallis test (due to the non-normal distribution of data based on the results of Kolmogorov–smirnov test), have been presented in Table 2. Most mosquitoes’ samples were collected from Silvana (44.851419, 37.428667) and the highest species richness was observed in Gojar (44.833728, 39.487847).

Also the results of Dunn-Bonferroni post hoc analysis revealed that the chloride content of the water collected from Hashiyeh Rood (Khoy: 44° 58′ N - 38° 32′ E) and Alkalinity of the water collected from Kooraneh (Urmia: 45° 2′ N - 37° 40′ E) were significantly higher than other localities (P < 0.05), however, there was no significant difference in Temperature, pH, Turbidity, TDS and EC among the different localities (P > 0.05) (Table 2).

Means and standard deviations of the physicochemical factors of the larval habitats of the different species have been shown in Table 3. The Kruskal–Wallis statistical test was used to examine the significance of the differences in each of the environmental factors in the different localities We found a significant difference in chloride content (*p Value*: 0.012) and temperature (*p Value*: 0.003) preference among the different species (*P *< 0.05), but the difference was not significant for pH (*p Value*: 0.576), Alkalinity (*p Value*: 0.622), Turbidity (*p Value*: 0.553), TDS (*p Value*: 0.572), and EC (*p Value*: 0.572) (*P *> 0.05).

**Table 3:** Means of physicochemical characteristics, along with occurrence of mosquito species in different larval habitats In West Azerbaijan province in 2018

	Species (number of occurrence)	*P* Value						
Physicochemical parameters (Mean ± SD)	*Cx. pipiens (15)*	*Cx. theileri (13)*	*Cx. mimeticus (6)*	*Cx. modestus (1)*	*Cs. longieraolata (5)*	*An. maculipennis (10)*	*Ae. Caspius (7)*	
pH	7.27 ± 0.46	7.31 ± 0.48	7.00 ± 0.00	7	7.20 ± 0.45	7.50 ± 0.71	7.57 ± 0.79	0.576
Temperature	25.13 ± 1.64	24.62 ± 1.71	23.00 ± 0.00	25	23.00 ± 0.00	24.30 ± 1.77	26.43 ± 1.13	0.003
Turbidity	3.73 ± 3.10	3.54 ± 3.23	1.50 ± 1.87	2	2.80 ± 2.59	2.40 ± 2.22	4.43 ± 4.08	0.553
Chloride	153.87 ± 153.87	88.85 ± 164.32	21.17 ± 19.78	295	28.40 ± 27.94	35.40 ± 31.83	205.57 ± 200.90	0.012
Alkalinity	285.40 ± 144.47	220.31 ± 96.97	237.33 ± 122.50	217	334.00 ± 168.75	325.00 ± 141.60	298.29 ± 148.35	0.622
TDS	505.40 ± 305.29	505.92 ± 254.31	379.83 ± 102.10	149	366.20 ± 108.68	473.10 ± 248.32	366.00 ± 285.30	0.572
EC	842.27 ± 508.77	843.00 ± 423.60	633.00 ± 170.10	249	610.40 ± 181.13	788.30 ± 413.70	610.00 ± 475.48	0.572

The affinity between the pairs of species *Cx. mimeticus* and *Cs. longiareolata* was 0.526, indicating a strong affinity. There was no affinity between the other pairs of species or the affinity was very weak; *Cx. mimeticus* and *Cs. longiareolata* (0.526), *Cs. longiareolata* and *An. maculipennis* (0.483), *Cx. theileri* and *An. maculipennis* complex (0.475), *Cx. pipiens* and *Ae. caspius* (0.456), *Cx. pipiens* and *Cx. theileri* (0.444), *Cx. mimeticus* and *An. maculipennis* complex (0.441), and *Cx. theileri* and *Cx. mimeticus* (0.427). The affinity indices between the pairs of species have been shown in Table 4.

**Table 4:** Percent of co-occurrence and affinity index between pairs of culicidae species in visited breeding places of the study area, west Azerbaijan province in Iran during 2018

Species	*Cx. pipiens*	*Cx. theileri*	*Cx. mimeticus*	*Cx. modestus*	*Cs. longieraolata*	*An. maculipennis*	*Ae. caspius*
*Cx. pipiens*	*	28.6	4.8	6.3	10	16	27.3
*Cx. theileri*	*0.444*	*	26.3	0	22.2	30.4	15
*Cx. mimeticus*	− 0 .024	*0.427*	*	0	36.4	31.3	0
*Cx. modestus*	0.129	− 0.139	− 0.204	*	0	0	12.5
*Cs. longieraolata*	0.102	0.357	***0.526***	− 0.5	*	33.3	0
*An. maculipennis*	0.197	*0.475*	*0.441*	− 0.5	*0.483*	*	11.8
*Ae. caspius*	*0.456*	0.176	− 0.204	− 0.122	− 0.224	0.081	*

The bold italic value indicates the highest affinity between the studied species (*Cs. longiareolata* and *Cx. mimeticus*)

The italic values of affinity between the pairs of species indicates the highest values between the studied pairs of species respectively

**Fig. 2:**
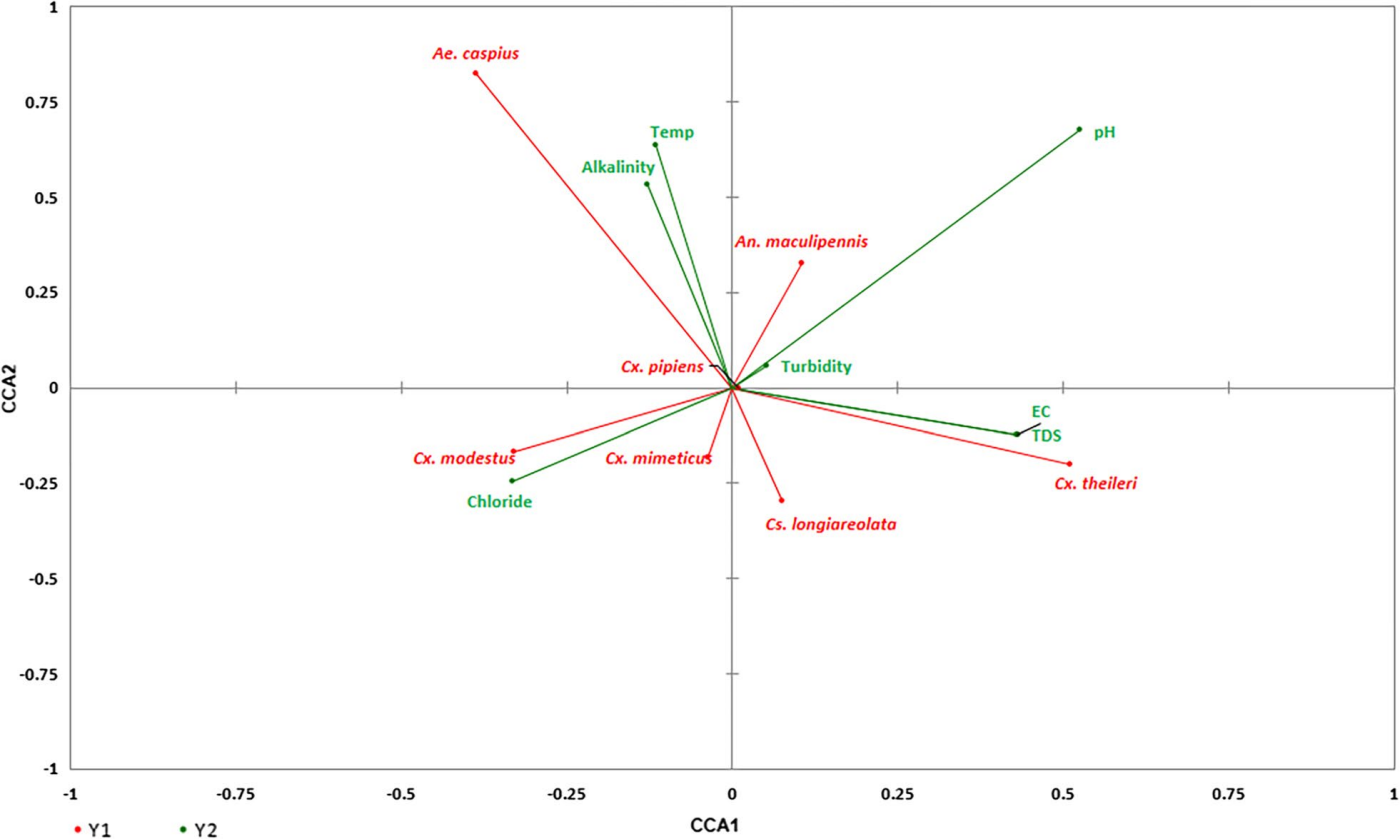
Biplot representing the results of CCA, the relationship between the presence of mosquito species (red arrows and labels) and the physicochemical variables (green arrows)

The species’ position in space relative to the main coordinates CCA1 and CCA2 and its relationship to the direction of the gradient of the physicochemical variables have been presented in Fig. 2. The constrained inertia for the model with these variables was 27.73% for CCA2 and 32.67% for CCA1. Species-physicochemical variables interaction for all 7 variables confirmed that pH and turbidity for *An. maculipennis*, Alkalinity and Temprature for *Ae. caspius*, Chloride for *Cx. mimeticus* and *Cx. modestus* and EC and TDS for *Cx. theileri* and *Cs. longiareolata* were the most important factors that positively and directly correlated with their presence and abundance (Fig. 2).

## Discussion

The effects of physicochemical factors of larval habitats on the distribution of mosquito larvae were examined for the first time in the west Azerbaijan Province, northwestern Iran. In this investigation, we tried to study the distribution and physicochemical factors of mosquito larval habitat in 5 counties of the West Azerbaijan Province, where various mosquito vectors of arboviruses are present. Some studies have been conducted on the fauna and checklist of mosquitoes in parts of this region [21, 38, 39].

Canonical correspondence Analysis (CCA) of the effects of the physicochemical factors on the mosquito species showed a correlation between the presence of *Cx. pipiens* and the studied environmental variables, which is consistent with the findings of a recent study which reported a significant positive correlation between the density of *Cx*. *pipiens* and electrical conductivity, alkalinity, total hardness and chloride [16].

Also, the correlation of the abundance of specific species with any physicochemical factors can be one of the reasons for the selective distribution of species in specific areas and the selection of suitable habitat for oviposition. Identifying the exact effect of physicochemical factors on the presence and abundance of mosquito larvae in different habitats seems to be a complex process and highly dependent on the characteristics of any specific species and related factors such as the pattern of chemical use in the environment (such as pesticides). Some of the physicochemical parameters could be used as a source of energy to expand the distribution of algae and other organisms (including bacteria) that function as key food for larvae [40], and to provide chemical stimuli for females to select a suitable oviposition site and trigger egg hatching [41].

However, the results of a recent study in Iran showed no significant correlation between the abundance of larvae and the studied different physicochemical and microbial parameters [15].

Water temperature acts as one of the most important factors affecting the establishment and growth of some of mosquito larvae in larval habitats [42]. Turbidity has also been reported to affect water temperature [43]. Based on the results of previous studies and due to the high correlation between some physicochemical factors and larval abundance of some species, it has been suggested that these factors be used as predictors of the presence of species in the environment, e.g. salinity and DO for *Culex pipiens* and *Cx. perexiguus* [9].

The distribution of mosquito species could be affected by various environmental factors such as physicochemical factors of their breeding places in the larvae stage, interspecific association, and climate [44].

In addition to biological curiosity and biodiversity, any change in the diversity and population of vector mosquitoes can affect the transmission cycle and burden of related diseases. As previously reported, global warming can increase the potential for transmission of mosquito-borne diseases [45]. Also previous studies have suggested that complex interactions exist between environmental factors and abundance of mosquito species, which necessitates time-dependent and species-specific control programs [46]. In particular, regular and accurate monitoring of the status of important medical mosquitoes, factors affecting their distribution and population, and prediction of their control strategies in specific conditions for sensitive region such as West Azerbaijan province is of particular importance, due to its border with four neighboring countries, presence of a variety of climatic conditions, and history of mosquito-borne diseases.

The species identified in this study can transmit important pathogens. Diseases such as lymphatic filariasis [47], and arboviruses [48] can be transmitted by different species of Culex mosquitoes. *Ae. caspius* infection with West Nile fever virus has already been demonstrated in our study area [25]. *An. maculipennis*, which is one of the important vectors of malaria [49], is of medical importance with a history of transmission in the region. Finally *Cs. longiareolata* acts as the vector of some infectious diseases such as the avian malaria [50, 51], tularemia [52], and arboviruses like West Nile fever [53–55].

A recent study [56, 57] reported the presence of five of the species identified in our study, including *An. maculipennis, Cx. pipiens, Cx. theileri*, *Cs. longiareolata* and *Ae. caspius* in the East Azerbaijan province. Also, another study reported the presence of *An. maculipennis, Cx.pipiens, Cx. theileri*, and *Cs. longiareolata*) in Zanjan Province, which were also identified in our study [58]. Moreover, another study conducted in Kurdistan Province [59] reported the occurrence of six species, including *An. maculipennis, Cx. theileri, Cx. pipiens, Cx. mimeticus, Cs. longiareolata,* and *Ae. caspius*, which were also present in our study region. In the Kurdistan and East Azarbaijan provinces, *Cx. theileri and Cx. pipiens* were the most dominant species, which is similar to the findings of the present study. Our results show that *Cx. theileri* (10 sites) and *Cx. pipiens* (13 sites) were the most dominant species in the province.

A significant difference was observed between the density of *An*. *culicifacies* and calcium and EC content, and between *An*. *turkhudi* and *An*. *superpictus* and total hardness in Sistan and Baluchestan Province of Iran [12]. The authors indicated that the larvae of *An*. *culicifacies* and *An*. *turkhudi* are more sensitive to physicochemical factors in different habitats compared with other species, which may explain the limited spread of the species in this region. Nikookar et al. [16] showed that positive correlation exists between the larval abundance of *Cx*. *pipiens* and physicochemical characteristics such as EC, alkalinity, total hardness, and chloride. However, Abai et al. [15] demonstrated that there was no significant differences in physicochemical and microbial parameter preferences among the different species, and that there was no significant correlation between the abundance of larvae and the different physicochemical and microbial parameters. Also for anopheline species, the significant effects of conductivity, total alkalinity, sulphate and chloride on their distribution and abundance have been reported [60].

Based on the results of the current study, there was a strong affinity between *Cx. mimeticus* and *Cs. longiareolata* (index˃0.5), but in a previous studies, no significant correlation between the presence of these two species was found [16, 61]. The difference in the results of the present study with the previous study may be due to differences in the specific environmental conditions of each region and the interaction of complex conditions in addition to the physicochemical properties of the environment with the mosquito larvae. Based on our affinity index analysis, there was no correlation between the other species which may be due to the different requirements of these species. In a recent study in southern Iran [4], a significant correlation between the following species was found: *An. Culicifacies/An. stephensi* and *An. dthali*, *An. dthali/An. stephensi* (Index > 0.5),. *An. dthali/An. superpictus* and *An. moghulensis*, and *An. moghulensis/An.superpictus*. Based on the affinity index of the above study, there was no correlation between *An. apoci* and other species.

Considering the limitations of this study, future studies should focus on a wider geographical area and the use of accurate methods for collecting mosquito larvae in order to provide evidence-based contributions to the integrated vector management.

As a limitation, in the current study, samples were collected from the same breeding site over time, pseudo-replication should be assumed and proper models such as the LMEM model should be used for future experiments to evaluate the fixed effect, the random effect and even the response variable. Also, using more accurate statistical analysis for evaluating the correlation between physicochemical factors and the presence and abundance of mosquitoes will lead to more valuable and accurate information.

## Conclusions

Due to the considerable climatic and environmental variability affecting the establishment and spread of mosquitoes (especially diseases’ vector species) in different regions of Iran, and in particular, in the study area, the results of the present study, will be useful in determining the environmental and biological factors of the larval habitats of medically important mosquitoes. Consequently, identification of environmental factors affecting the breeding of mosquitoes can help in the mapping and prediction of oviposition site selection and distribution of these vectors and pave way for the development of entomological monitoring and targeted control programs for controlling the vectors with higher accuracy.

## Data Availability

The datasets used and/or analyzed during the current study are available from the corresponding author on reasonable request.

## Abbreviations

NTU: Turbidity
TDS: Total Dissolved Solids
EC: Electrical Conductivity
pH: Acidity
